# Increase in *Neisseria meningitidis* Serogroup W135, Niger, 2010

**DOI:** 10.3201/eid1609.100510

**Published:** 2010-09

**Authors:** Jean-Marc Collard, Zaneidou Maman, Harouna Yacouba, Saacou Djibo, Pierre Nicolas, Jean-François Jusot, Jocelyne Rocourt, Rabi Maitournam

**Affiliations:** Author affiliations: Centre de Recherche Médicale et Sanitaire, Niamey, Niger (J.M. Collard, S. Djibo, J. Rocourt, J.F. Jusot);; Ministère de la Santé Publique, Niamey (Z. Maman, H. Yacouba, R. Maitournam);; Institut de Médecine Tropicale du Service de Santé des Armées, Marseille, France (P. Nicolas)

**Keywords:** Neisseria meningitidis, bacteria, serogroup W135, meningitis, Niger, outbreak, vaccination, surveillance, letter

**To the Editor:** Meningococcal epidemics in the African meningitis belt are generally caused by *Neisseria meningitidis* serogroup A strains, but they also can be caused by serogroup W135 or X strains. The largest reported outbreak caused by serogroup W135 occurred in Burkina Faso in 2002 with ≈13,000 suspected cases (*1*). Sporadic cases of meningitis caused by serogroup W135 have, however, been detected previously, notably in Niger since the early 1980s (*2*). This serogroup has also been associated with outbreaks in pilgrims to Mecca, Saudi Arabia, in 2000, and several clusters of cases occurred worldwide before 2002 (*3*). After 2003, no major outbreak caused by serogroup W135 was detected in sub-Saharan countries, only sporadic cases. Although Niger borders Burkina Faso, Niger has not experienced a large outbreak of meningitis caused by serogroup W135, with the exception of 7,906 suspected cases and 595 deaths declared in 2001; serogroup W135 represented 12 (38.7%) of the small number (n = 31) of confirmed cases (*4*). In 2010, serogroup W135 may have caused a major outbreak (a large proportion of this serogroup was detected during the first 12 weeks). Niger residents have not been in contact with this serogroup in recent years and have never been immunized with the trivalent polysaccharide vaccine (A/C/W135).

From January 1 through March 28, 2010, the Ministry of Public Health of the Republic of Niger reported 1,188 suspected cases of meningococcal disease, including 103 deaths (case-fatality rate 8.7%). Suspected cases were reported from all 8 provinces but predominantly in the provinces of Maradi (40%) and Tillabéry (24%). At week 12, the districts of Maradi Commune and neighboring Madarounfa crossed the alert, or epidemic, threshold with cumulated attack rates per 100,000 inhabitants of 57.0 and 48.5, respectively. Zinder City district also crossed the alert threshold.

Laboratory confirmation and microbiologic surveillance of meningococcal meningitis is conducted by the Centre de Recherche Médicale et Sanitaire by using culture or PCR (*5*) techniques on cerebrospinal fluid (CSF) or CSF-inoculated trans-isolates. During the study period, the Centre received 816 CSF or trans-isolate specimens (from 69% of the notified cases). Culture (n = 23, 2.8%) and PCR (all specimens) identified *N. meningitidis* as the predominant pathogen (n = 248, 30.4%), followed by *Streptococcus pneumoniae* (n = 35, 4.3%) and *Haemophilus influenzae* (n = 13, 1.6%). Among the 248 cases with confirmed meningococcal etiology, the most frequent serogroup was W135 (n = 121, 48.8%), followed by A (n = 116, 46.8%) and X (n = 2), indicating that serogroup W135 had increased markedly compared with the past 2 years (Figure). Among the 816 CSF specimens, 454 (56%) remained negative when tested for the presence of *N. meningitidis*, *S. pneumoniae*, or *H. influenzae* by PCR. Eighty-four (69.4%) of the serogroup W135 strains originated from the province of Maradi (southern Niger) and, more specifically, 36%.4% (n = 44) and 19.8% (n = 24) originated from the Madarounfa and Maradi districts, respectively. In contrast, serogroup A was mainly present in Tillabéry (western Niger) with 49.1% (n = 57) of the strains and, to a lesser extent, in the provinces of Maradi (16.4%, n = 19) and Dosso (13.8%, n = 16). All meningococcal strains (n = 9 for W135, n = 1 for A) recovered from trans-isolates and analyzed by Etest (AB bioMérieux, Marcy l’Etoile, France) were susceptible to beta-lactams (penicillin, amoxicillin, and ceftriaxone), chloramphenicol, and rifampin. This finding supports the appropriateness of World Health Organization recommendations for antimicrobial drug treatment. The A strain belonged to the sequence type (ST) 7 and the W135 strains to ST 11, the same ST of the strain associated with outbreaks in pilgrims in Saudi Arabia in 2000 (*3*) and the strain that caused the large epidemic in Burkina Faso in 2002 (*1*).

**Figure:**
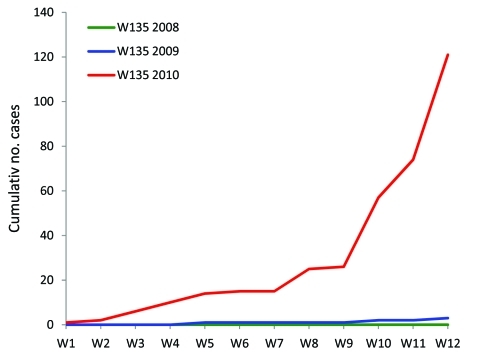
Epidemic curve of cumulative confirmed cases of *Neisseria meninigitidis* serogroup W135 infections, Niger, 2008, 2009, and 2010 (weeks 1–12). No cases were found in 2008.

The mean ages of patients with confirmed cases of infection with serogroup W135 and serogroup A were 8.1 (SD 8.5) and 10.9 (SD 7.9) years, respectively. Although no significant difference was found in the mean ages, the age group was 1–4 years of age had more disease caused by serogroup W135, and children 5–14 years of age were most affected by serogroup A. Similarly, the attack rate during the outbreak of meningitis caused by serogroup W135 in Burkina Faso in 2002 was highest in patients <5 years of age, and the attack rate decreased as patients’ ages increased (*6*).

Reactive vaccination campaigns in some communes of Madarounfa district that had reached the epidemic threshold were launched by the Ministry of Public Health with a remaining 2009 stockpile (16,527 doses, 35.7% coverage) of the quadrivalent polysaccharide vaccine (A/C/Y/W135) from Médecins sans Frontières. The International Coordinating Group on Vaccine Provision for Epidemic Meningitis Control has also recently approved the release of 381,526 doses of trivalent polysaccharide vaccine (A/C/W135) for vaccination campaigns in Maradi and Zinder districts. Future immunization campaigns will be implemented by Ministry of Public Health with the support of the World Health Organization and partners, including Médecins sans Frontières and The United Nations Children’s Fund.

Given the large population at risk, and the low availability and high cost of the trivalent vaccine, a sound vaccination strategy is of particular importance to mitigate the expansion of serogroup W135 in the country. Microbiologic surveillance is critical in the early and accurate detection of meningococcal serogroups for determining the appropriate vaccine.

## References

[1] World Health Organization. Meningococcal meningitis. Wkly Epidemiol Rec. 2003;78:294–6. 14509123

[2] Denis F, Rey J-L, Amadou A, Saliou P, Prince-David M, M’Boup S, Emergence of meningococcal meningitis caused by W135 subgroup in Africa. Lancet. 1982;2:1335–6. 10.1016/S0140-6736(82)91533-1 6128617

[3] Borrow R. Meningococcal disease and prevention at the Hajj. Travel Med Infect Dis. 2009;7:219–25. 10.1016/j.tmaid.2009.05.003 19717104

[4] Taha MK, Parent Du Chatelet I, Schlumberger M, Sanou I, Djibo S, de Chabalier F, *Neisseria meningitidis* serogroups W135 and A were equally prevalent among meningitis cases occurring at the end of the 2001 epidemics in Burkina Faso and Niger. J Clin Microbiol. 2002;40:1083–4. 10.1128/JCM.40.3.1083-1084.2002 11880446 PMC120283

[5] Chanteau S, Sidikou F, Djibo S, Moussa A, Mindadou H, Boisier P. Scaling up of PCR-based surveillance of bacterial meningitis in the African meningitis belt: indisputable benefits of multiplex PCR assay in Niger. Trans R Soc Trop Med Hyg. 2006;100:677–80. 10.1016/j.trstmh.2005.09.006 16359713

[6] Nathan N, Rose AMC, Legros D, Tiendrebeogo SRM, Bachy C, Bjrlw E, Meningitis serogroup W135 outbreak, Burkina Faso, 2002. Emerg Infect Dis. 2007;13:920–3. 17553237 10.3201/eid1306.060940 PMC2792856

